# A Case of Pancreatic Pseudocyst Complicated by Pseudoaneurysm

**DOI:** 10.7759/cureus.2512

**Published:** 2018-04-20

**Authors:** Ashley Goordeen, Mohamad Sharbatji, Sameen Khalid, Aamer Abbass, Umair Majeed

**Affiliations:** 1 University of Central Florida College of Medicine, Orlando, USA; 2 Internal Medicine, Florida Hospital-Orlando, Orlando, USA; 3 Internal Medicine Residency, Florida Hospital-Orlando, Orlando, USA

**Keywords:** pancreatitis, pancreatic pseudocyst, chronic pancreatitis, pseudo-aneurysm

## Abstract

Pancreatic pseudocyst is a complication that can arise in both acute and chronic pancreatitis. Overtime, this encapsulated enzyme-rich fluid collection may erode into surrounding vasculature and result in the formation of a pseudoaneurysm. Pseudoaneurysms can rupture into the gastrointestinal tract and present as upper, lower, and biliary bleeding. Evaluation of pancreatic pseudocysts involves computed tomography imaging or magnetic resonance imaging for both identification and monitoring. Esophagogastroduodenoscopy (EGD) and endoscopic ultrasound (EUS) can be done to further visualize the lesion. In the presence of gastrointestinal bleed, management involves the combination of interventional radiology and surgery.

## Introduction

A pancreatic pseudocyst is an encapsulated enzyme-rich fluid collection with a well-demarcated wall of inflammation that can form after four weeks following acute pancreatitis or in chronic pancreatitis. Risk factors for pancreatitis include excessive alcohol intake, hypertriglyceridemia, biliary tract disease, trauma, viral infections, drugs, and genetic mutations. Though the incidence of a pancreatic pseudocyst is low, it is usually fatal due to high risk of erosion into surrounding vasculature resulting in the formation of a pseudoaneurysm. The majority of reported cases show pseudoaneurysm formation in the splenic artery, gastroduodenal artery, and anterior pancreaticoduodenal artery [1]. This pseudoaneurysm can rupture into various areas of the gastrointestinal tract such as the stomach, pancreatic duct, bile duct, duodenum or colon. This can present as upper, lower, or biliary tree gastrointestinal bleeding [2]. Patients with pancreatic pseudocysts may be asymptomatic or complain of non-specific symptoms such as persistent abdominal pain or loss of appetite [3]. The presence of an infected pseudocyst may produce jaundice or sepsis. On physical examination, there may be abdominal tenderness and a palpable abdominal mass. Computed tomography (CT) imaging will reveal a well circumscribed, round or oval, homogeneous density. We report a case of severe upper gastrointestinal bleed from a fistulizing hemorrhagic pancreatic pseudocyst into the stomach.

## Case presentation

**Figure 1:**
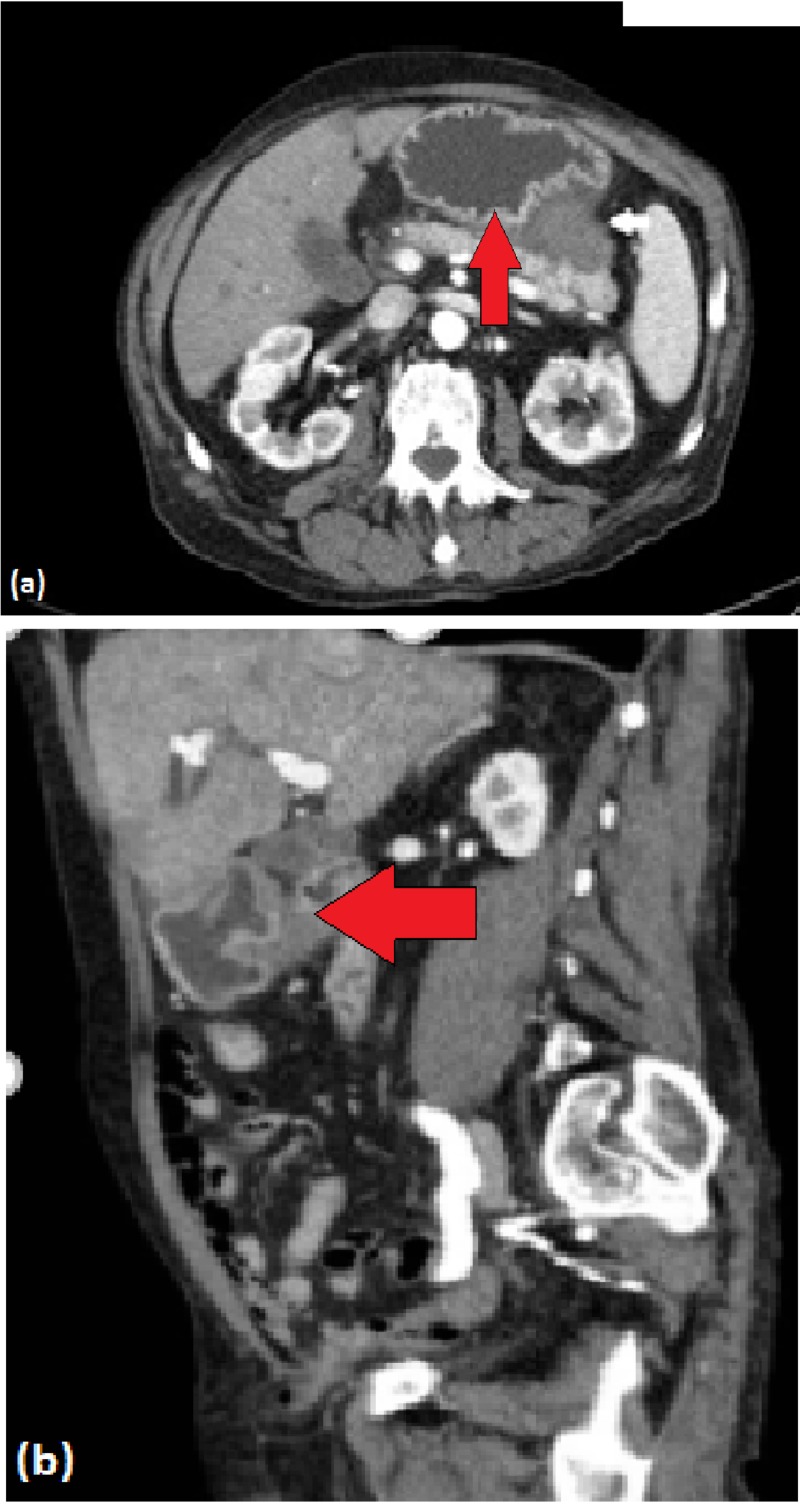
Computed tomography of complex pancreatic mass measuring 5.5 x 4.9 x 4.3 cm, which asserts mass effect along the proximal greater curvature of the stomach towards the fundus (red arrows). (a) Axial view. (b) Coronal view.

A 79-year-old male with a past medical history significant for pancreatic pseudocyst secondary to idiopathic acute pancreatitis diagnosed two years ago presents with severe acute gastrointestinal bleed with a hemoglobin of 5.8 g/dL. Throughout the week prior to admission, the patient had been experiencing melena. He was admitted to the intensive care unit for supportive care including pantoprazole infusion, blood transfusion and close monitoring. CT scan of the abdomen revealed a complex pancreatic mass representing a bleeding pancreatic pseudocyst with an interval increase in size when compared with the previous CT scans (Figure 1).

**Figure 2:**
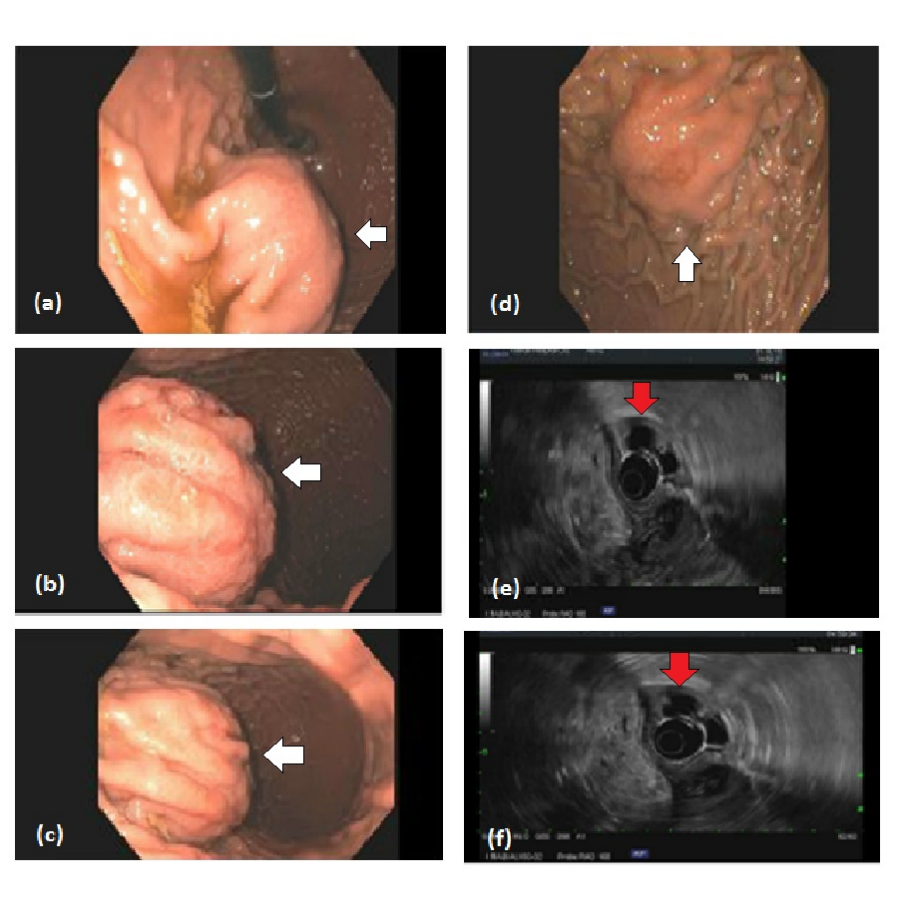
Esophagogastroduodenoscopy (EGD)/endoscopic ultrasound (EUS) of pancreatic pseudocyst. (a-d) EGD demonstrating submucosal bulge from extrinsic compression on the stomach in the proximal gastric body and fundus, there is also small clean-based ulcer on the top of this bulge (white arrows). (e-f) EUS showing anechoic lesion with hyperechoic shadowing suggestive of pseudocyst with bleeding that was identified in the pancreatic tail (red arrows).

The patient underwent esophagogastroduodenoscopy (EGD)/endoscopic ultrasound (EUS) which showed submucosal bulging likely from extrinsic compression on the proximal gastric body and fundus with small clean-based ulcer on the top of this bulge (Figure 2). No fresh or old blood was noted in the stomach, and the esophagus and duodenum both appeared normal. EUS confirmed the presence of anechoic lesion with hyperechoic shadowing suggestive of cyst/pseudocyst with bleeding located in the tail of the pancreas. The lesion measured approximately 50 x 50 mm in the maximal cross-sectional diameter.

**Figure 3:**
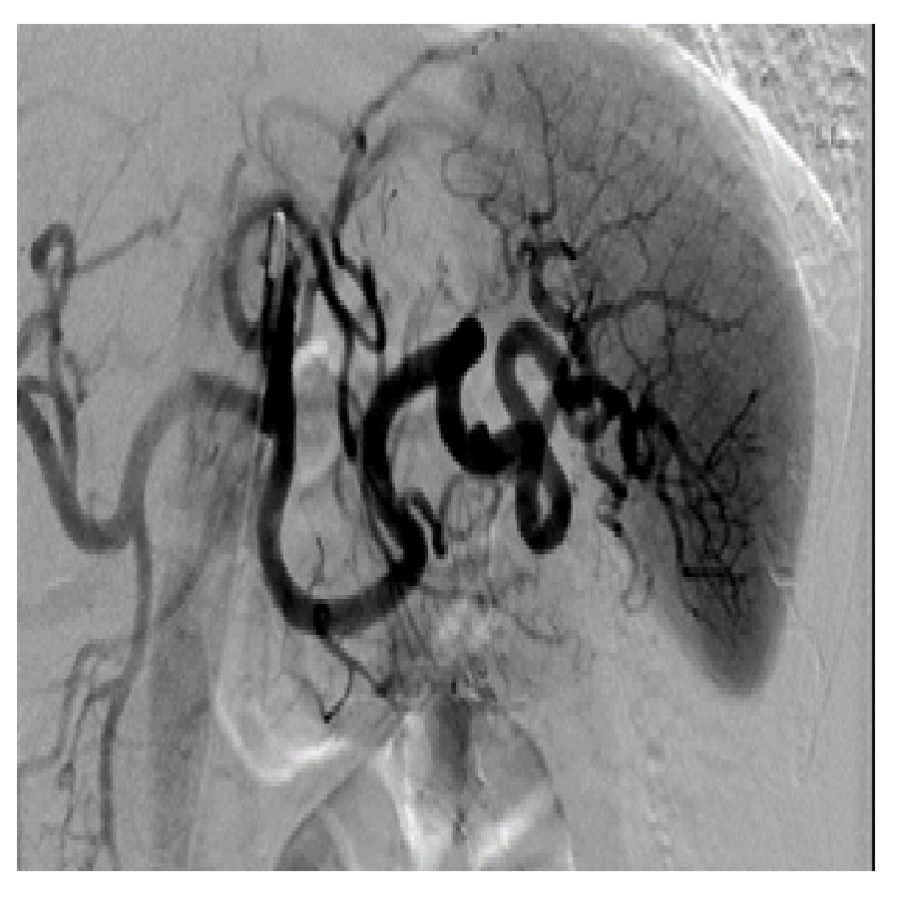
Computed tomography angiogram of celiac artery showed no evidence of active pseudocyst bleeding or vascular abnormality.

**Figure 4:**
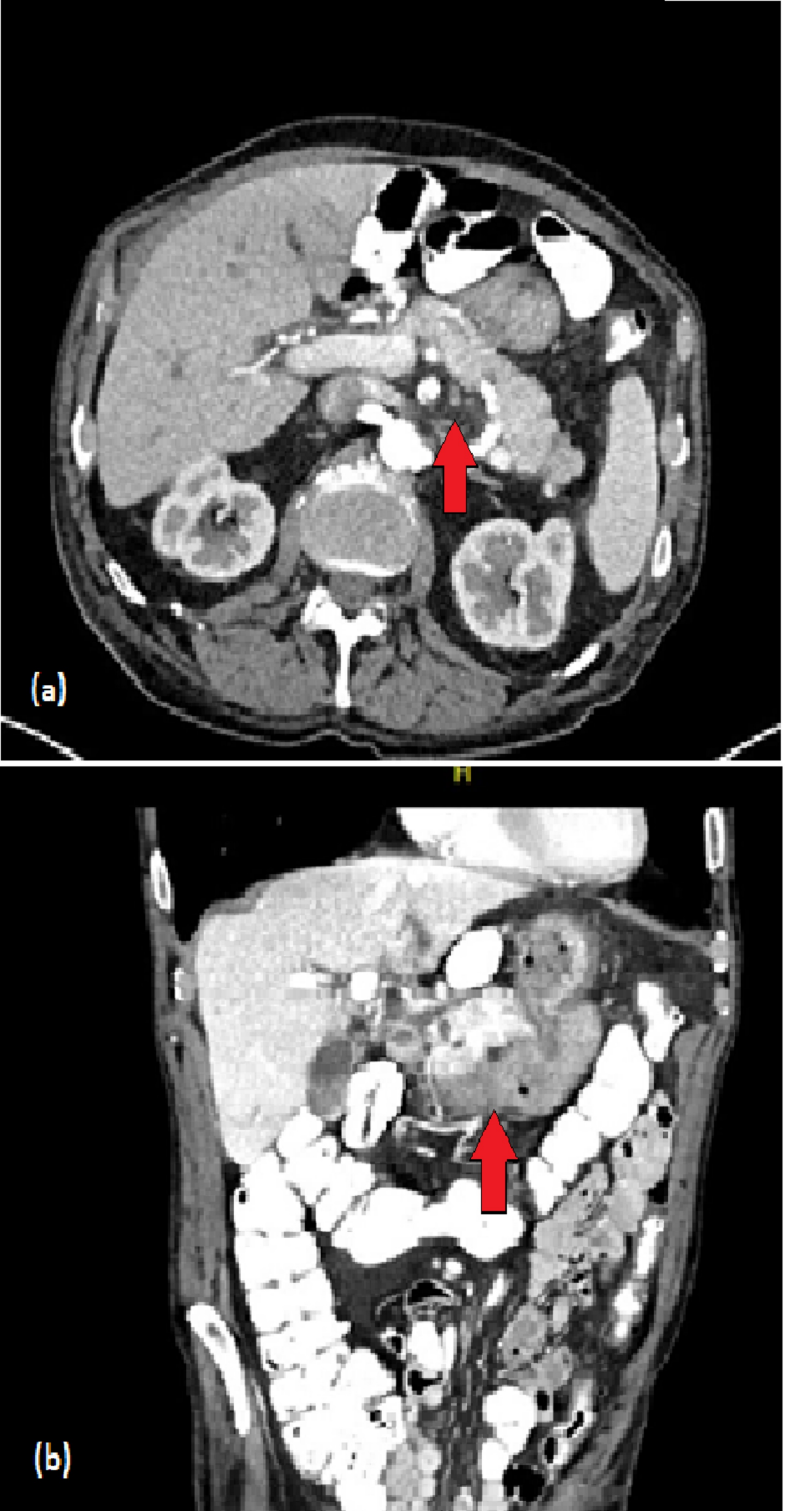
Follow-up computed tomography abdomen. Findings consistent with chronic pancreatitis within the head/uncinate of the pancreas (red arrows). The cystic lesions in the head and tail of the pancreas have continued to decrease in size. The pseudocyst abutting the wall of the stomach has resolved. (a) Axial view. (b) Coronal view.

The patient continued to have melena and a decrease in hemoglobin to 6.3 g/dL for which an additional two units of packed red blood cells (PRBCs) were provided. Interventional radiology was asked to perform mesenteric angiogram with hope that may help identify and control the source of bleeding, however, angiogram did not demonstrate any active bleeding vessel (Figure 3). Ultimately, the bleeding stopped spontaneously. The patient had another EGD/EUS which again noted two openings over the greater curvature of the stomach suggestive of fistulous communication of the lesion with the stomach lumen. A bullet-tipped catheter was inserted into the lumen and aspiration showed blood consistent with hemorrhagic pancreatic pseudocyst fistulizing into the stomach and causing severe upper gastrointestinal bleed. Follow-up CT abdomen one year later revealed calcification in the head and uncinate process of pancreas along with scarring in the uncinate process, consistent with chronic pancreatitis (Figure 4). Additionally, the pseudocyst that was abutting the stomach wall had resolved.

## Discussion

Pancreatic pseudocysts are a sequela known to occur following acute and chronic pancreatitis. These enzyme-rich fluid-filled collections are well-defined and encapsulated. The incidence of pseudocysts is 5-16% in acute pancreatitis and as high as 20-40% in chronic pancreatitis [4]. Complications associated with pancreatic pseudocysts are infection, rupture and hemorrhage. They can erode into adjacent vessels, the splenic artery most commonly, and produce a pseudoaneurysm. These pseudoaneurysms can rupture and result in intraperitoneal and gastrointestinal bleed. Patients are at risk of rebleeding days to years following their initial episode of bleeding with increased mortality if not properly managed [5].

Spontaneous resolution (8-70%) of pancreatic pseudocyst depends on numerous factors such as size, chronicity, etiology, and thickness of its wall [6]. Conservative management entails the use of analgesics, intravenous fluids and antiemetics as supportive therapy for symptomatic patients. Low-fat diets are recommended for patients that can tolerate oral intake, while those that cannot can receive support through nasoenteral feeds or total parenteral nutrition. Pseudocysts complicated by pseudoaneurysm rupture are managed using a combination of interventional radiology and surgery.

Hemodynamically stable patients initially undergo CT angiogram to confirm the diagnosis. Interventional radiology can then be used to embolize the offending vessel or a stent may be placed if visceral ischemia is a concern [5]. Unstable patients may require surgical intervention consisting of resection of the pseudocyst and pseudoaneurysm. Drainage of the pseudocyst and proximal and distal ligation of the artery containing the pseudoaneurysm is another technique that may be utilized.

## Conclusions

Acute and chronic pancreatitis may be associated with the formation of pancreatic pseudocysts. This complication can pose lethal consequences in patients not treated properly. Erosion of the cysts into nearby vasculature can lead to the development of a pseudoaneurysm that may result in hemorrhage. Proper management of pancreatic pseudoaneurysm hemorrhage involves the use of vessel embolization or stenting and offending artery ligation depending on patient hemodynamic stability.
